# Computational investigation of dynamical transitions in Trp-cage miniprotein powders

**DOI:** 10.1038/srep25612

**Published:** 2016-05-06

**Authors:** Sang Beom Kim, Devansh R. Gupta, Pablo G. Debenedetti

**Affiliations:** 1 Department of Chemical and Biological Engineering, Princeton University, Princeton, New Jersey 08544, United States

## Abstract

We investigate computationally the dynamical transitions in Trp-cage miniprotein powders, at three levels of hydration: 0.04, 0.26 and 0.4 g water/g protein. We identify two distinct temperatures where transitions in protein dynamics occur. Thermal motions are harmonic and independent of hydration level below *T_low_* ≈ 160 K, above which all powders exhibit harmonic behavior but with a different and enhanced temperature dependence. The second onset, which is often referred to as the protein dynamical transition, occurs at a higher temperature *T*_*D*_ that decreases as the hydration level increases, and at the lowest hydration level investigated here (0.04 g/g) is absent in the temperature range we studied in this work (*T* ≤ 300 K). Protein motions become anharmonic at *T*_*D*_, and their amplitude increases with hydration level. Upon heating above *T*_*D*_, hydrophilic residues experience a pronounced enhancement in the amplitude of their characteristic motions in hydrated powders, whereas it is the hydrophobic residues that experience the more pronounced enhancement in the least hydrated system. The dynamical transition in Trp-cage is a collective phenomenon, with every residue experiencing a transition to anharmonic behavior at the same temperature.

Although proteins are often described with static representations obtained from X-ray crystallographic data, the dynamics of proteins are essential to their functionality, as is clearly illustrated, for example, in the case of ligand binding and ion channel regulation^1^,^2^. The characteristic amplitude of protein motions, often measured by atomistic mean-square fluctuations (MSF) of the protein atoms, increases linearly with temperature up to approximately 180–240 K^3^,^4^. At this temperature the amplitude exhibits a sharp transition to a non-linear temperature dependence, and this onset of anharmonicity is referred to as the protein dynamical transition (PDT)^3^. Experimental studies report impaired protein functionality at temperatures below the dynamical transition temperature (*T*_*D*_)^5^,^6^,^7^.

For a given protein, the PDT shifts to higher temperatures with increasing solvent viscosity, which is sensitive both to hydration level and solvent composition^8^,^9^,^10^,^11^,^12^. Despite many experimental and computational studies^4^,^8^,^9^,^10^,^11^,^12^,^13^,^14^,^14^,^15^,^16^,^17^,^18^,^19^,^20^, the underlying physical basis of the PDT is still under debate. Some consider the main contribution to this transition to come from the activation of protein side-chain dynamics due to the increased translational and rotational dynamics of water^14^,^17^,^18^. It has also been suggested that the PDT is connected to the fragile-to-strong transition of the hydration water and the hypothesized liquid-liquid transition, and corresponds to the crossing of the Widom line^19^,^20^.

It has been found that there exists another transition in protein dynamics at a temperature lower than *T*_*D*_^4^,^13^,^14^,^15^,^16^. Protein dynamics exhibit an onset of enhanced motion at this transition temperature, which we refer to as *T*_*low*_ in this paper. The *T*_*low*_ is known to be dependent on the type of protein, typically ranging from 100 K to 180 K^21^,^22^,^23^. The activation of methyl group motions^21^,^22^ and proline puckering transitions^23^ have been suggested as the underlying causes for this transition, but as with the PDT, the physical origin of this transition remains unclear.

In order to obtain atomic-level physical insight into these low-temperature transitions, we present a simulation study of protein powder systems with varying degrees of hydration level. Molecular dynamics (MD) simulations provide the appropriate spatial and temporal resolution to probe protein dynamics on a microscopic scale, complementing experimental studies. As a model protein, we used a 20-residue miniprotein Trp-cage, which is one of the smallest synthetic peptides that show protein-like secondary and tertiary structures (PDB ID: 1L2Y)^24^. At ambient conditions, Trp-cage has a well-defined hydrophobic core with both *α*-helix and 3_10_-helix structures^24^. The small size of Trp-cage makes it an ideal candidate for our study, where the simulations of multiple Trp-cages in a unit cell are needed to model powder-like environments. We chose to perform simulations of such an environment, rather than of a system composed of a single protein unit in solution because the powder system provides more information on protein dynamics directly relevant to practical applications, such as solid-state pharmaceutical formulations^25^,^26^.

The powder systems, each comprised of 16 Trp-cages, were prepared at three different hydration levels. We refer to these systems as P–0.40, P–0.26, and P–0.04 in this paper, where the number denotes the hydration level in g water/g protein (g/g). The three hydration levels were chosen to represent fully hydrated, partially hydrated, and dehydrated powders. Trp-cage is fully hydrated at a hydration level of 0.40 g/g, according to its water sorption isotherm^26^, and 0.04 g/g represents the approximate amount of residual, strongly bound water present in typical freeze-dried protein powders^27^. We systematically identify the two transitions and their dependence on the hydration level. We also show the effect of local hydrophobicity on residue-level protein dynamics.

## Results

**Figure 1:**
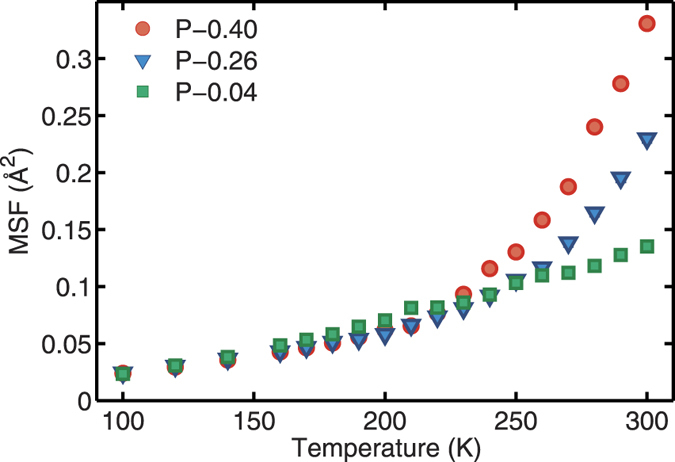
Average MSF as a function of temperature, at three different hydration levels. At temperatures below 150 K, the average MSF values are identical despite the different hydration levels. The error bars are smaller than the symbol sizes.

Figure 1 shows the average mean-square fluctuation (MSF) of protein heavy (non-hydrogen) atoms as a function of temperature, for each hydration level. Below ≈ 150 K, the protein dynamics are essentially harmonic, exhibiting a linear increase in the average MSF with temperature. In this range of temperatures, the magnitudes of the average MSFs are essentially identical among all hydration levels considered here. Thus, the average MSFs of the protein heavy atoms are not affected by the level of hydration at temperatures below *T*_*low*_.

**Figure 2:**
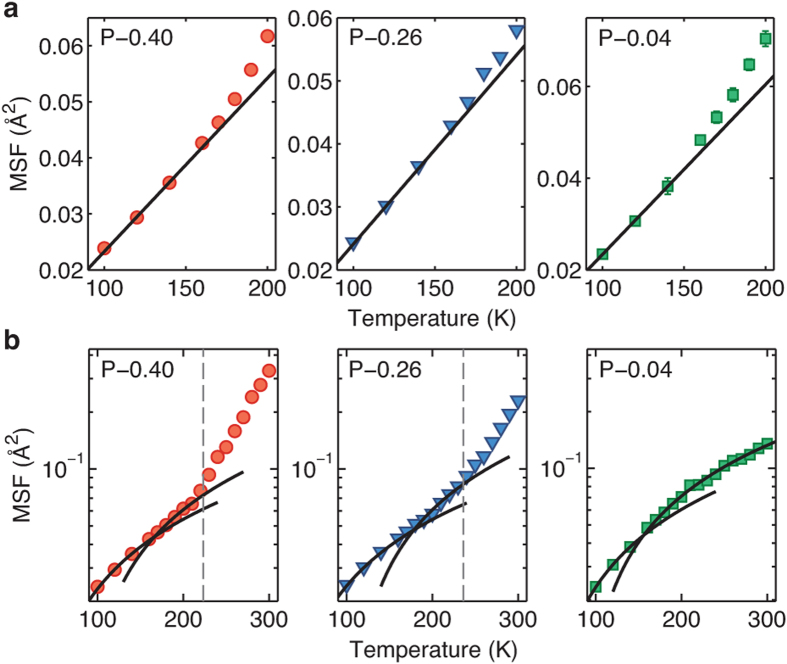
MSF as a function of temperature. (**a**) The MSF deviates from its low-temperature linear increase at ≈ 160 K (*T*_*low*_). The solid line is the linear fit to the temperature range between 100 K and 150 K. (**b**) The semi-logarithmic plots of the average mean-square fluctuation as a function of temperature. The solid lines represent the linear fits to the data in the temperature range below *T*_*low*_ and between *T*_*low*_ and 210 K. The dashed line shows the *T*_*D*_, at which the nonlinear increase in MSF begins. The error bars are shown or smaller than the symbol sizes.

Figure 2a shows the average MSFs in the temperature range *T* =  100–200 K, with linear fits to the 100–150 K regions. At *T* ≈  160 K, the average MSF starts deviating from the low-temperature linear increase with temperature. This temperature corresponds to the first transition temperature (*T*_*low*_), and the powders with varying hydration levels have a common value of *T*_*low*_, within the numerical accuracy of our calculations. The independence of *T*_*low*_ on hydration level is consistent with previous studies^21^,^28^.

Figure 2b shows the average MSF in semi-logarithmic scale as a function of temperature. The two solid lines represent linear fits to the data below *T*_*low*_ (≈ 160 K) and between *T*_*low*_ and 210 K. This indicates that the Trp-cage powders exhibit a linear T-dependence of the average MSF above *T*_*low*_ as well, but with an increased slope. With the exception of P–0.04 system that shows no PDT up to 300 K, the average MSF then starts to increase nonlinearly at *T* ≈  220–240 K. We refer to this temperature as the *T*_*D*_. Above *T*_*D*_, the amplitude of protein motions is significantly enhanced with increasing hydration level.

**Figure 3:**
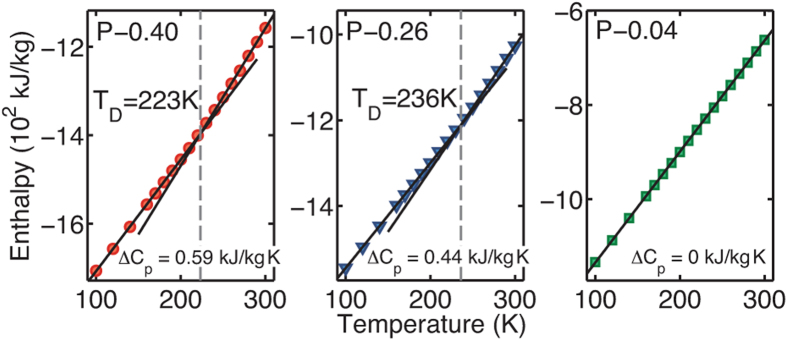
Jump in heat capacity at the dynamical transition temperature. Except for the P–0.04 system, two best-fit lines exist. Their intersection corresponds to *T*_*D*_. As the hydration level decreases, the *T*_*D*_ is shifted to a higher temperature while Δ *C*_*P*_ decreases. The error bars are smaller than the symbol sizes.

It can be nontrivial to determine *T*_*D*_ by locating the temperature at which the MSF starts increasing nonlinearly, especially for less-hydrated systems. This is because the onset of the anharmonic increase in dynamics becomes less pronounced as the hydration level decreases. It has been suggested that the PDT is related to the glass transition of the partially hydrated protein system^29^. Accordingly, we investigate the temperature dependence of the enthalpy, which should, according to this hypothesis, exhibit a discontinuous change in slope at *T*_*D*_, corresponding to a jump in the heat capacity^30^. Figure 3 shows the enthalpy of each powder system as a function of temperature. With the exception of the dehydrated system (P–0.04), two linear regimes with different slopes are indeed present. The question of whether this change of slope corresponds to a true glass transition is one that we do not address here; we simply note that this particular aspect of glassy behavior^30^ is present and, remarkably, we find these effective glass transition temperatures to be in very good agreement with the *T*_*D*_ found in Fig. 2b (dashed lines). *T*_*D*_ shifts to higher temperatures as the protein becomes dehydrated, confirming for the Trp-cage powders investigated here the experimentally observed dependence of the PDT upon the hydration level^8^,^9^,^10^,^11^,^12^. The marginally hydrated, P–0.04 system, exhibits a single linear regime for the enthalpy as a function of *T*, due to the lack of the PDT up to the highest temperature investigated here, *T* =  300 K. Finally, we note that the heat capacity jump, Δ *C*_*p*_, increases with the hydration level.

**Figure 4:**
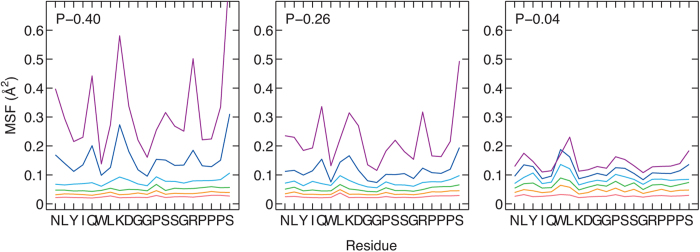
The MSF of each residue in Trp-cage. From bottom to top, the red, orange, green, cyan, blue, and violet lines represent the MSFs at 100, 140, 180, 220, 260 and 300 K. The hydrated (P–0.40 and P–0.26) and dehydrated (P–0.04) powders differ in the residues that become activated.

In order to understand how each residue contributes to the overall dynamics of the Trp-cage powders, we show the MSF of each residue in Fig. 4. It can be seen that certain residues exhibit especially high MSF values compared to the rest. The termini residues (N1 and S20) have high MSF values because they are only bonded to one other residue and thus experience comparatively less restraints. In the hydrated powders (i.e. P–0.40 and P–0.26), the non-terminus residues with high MSF values (Q5, K8, D9, S13 and R16) have the common trait of being hydrophilic, due to either nonzero charges (K8, D9, and R16) or polar functional groups that can form hydrogen bonds with water (Q5 and S13).

**Figure 5:**
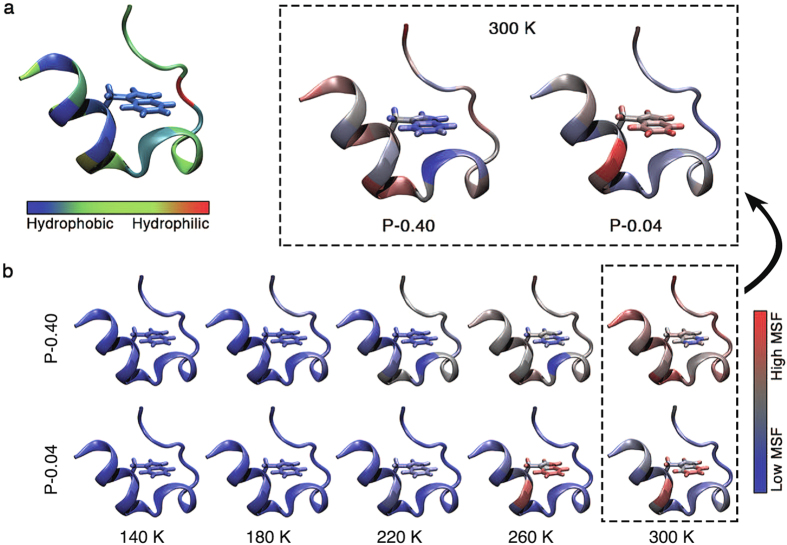
Colored visualizations of the localized dynamics of the Trp-cage. (**a**) The structure is colored based on the hydrophobicity scale of each amino-acid residue^31^. (**b**) Colors represent the MSF of each residue at 140, 180, 220, 260 and 300 K for the hydration levels of 0.40 and 0.04 g/g. The increasing magnitude of fluctuations is represented by the change in the color from blue to gray to red. The side chain of the Trp-6 residue is shown explicitly, because the burial of this hydrophobic residue plays a key role in the folding of the Trp-cage. The hydrophobic residues, including Trp-6, show very small MSFs even at 300 K in the hydrated system (P–0.40), while the opposite is observed in the dehydrated system (P–0.04). Note that for a clearer visualization the colors scale differently with the MSF values for the two hydration levels. The schematics at 300 K are enlarged and rescaled in color for a clearer contrast between the two systems.

The dehydrated system (i.e. P–0.04), however, exhibits opposite behavior. Figure 5a shows the Trp-cage unit, with each residue colored according to the hydrophobicity scale of Eisenberg *et al.*^31^. Figure 5b compares the powders with hydration levels 0.4 and 0.04 g/g by coloring each residue according to the magnitude of its MSF. As the temperature is increased, the MSF of the hydrophilic residues increases relative to that of the hydrophobic residues in the hydrated powder. In contrast, it is the hydrophobic residues (e.g. L2, W6 and L7) of the dehydrated powder that experience enhanced MSF upon heating. This enhancement of fluctuations in the hydrophobic residues suggests that dehydration can render the protein structure unstable by disrupting the burial of hydrophobic residues.

**Figure 6:**
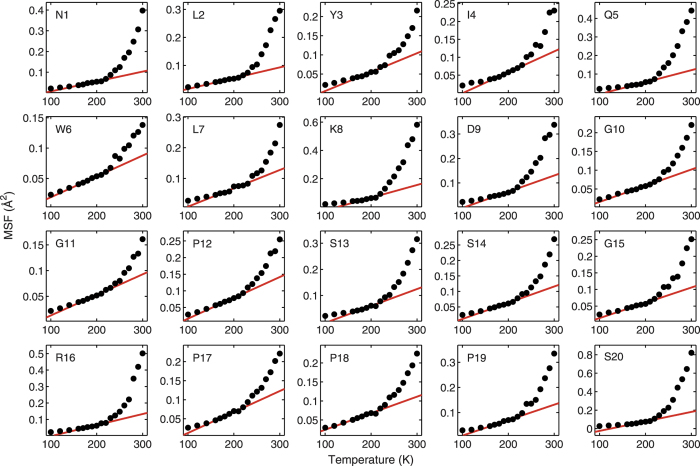
Average MSF of individual residues in Trp-cage (P–0.40 system). The red line depicts the linear fit to the data between temperatures *T*_*low*_ and *T*_*D*_ (160 K and 223 K, respectively). For all 20 residues, a slope change between two harmonic regimes occurs at *T*_*low*_ (160 K), while the onset of nonlinear increase starts approximately at *T*_*D*_ (223 K). The hydrophilic residues (e.g. K8, D9 and R16) exhibit larger-amplitude fluctuations for *T* >  *T*_*D*_, compared to the hydrophobic residues. The error bars are smaller than the symbol sizes.

In order to understand whether each residue exhibits the same abrupt transitions in the temperature dependence of its MSF as the protein as a whole, the average MSF of each residue in the P–0.40 system is plotted as a function of temperature in Fig. 6. Using the independently-determined *T*_*low*_ and *T*_*D*_ values for the entire protein (Figs 2 and 3), Fig. 6 shows individual linear fits to the MSF(*T*) data for each residue, with fits performed for *T*_*low*_ ≤  *T* ≤  *T*_*D*_. It can be seen that, within the numerical accuracy of the data, all 20 residues exhibit two transitions, at the same temperatures, *T*_*low*_ and *T*_*D*_, as the whole Trp-cage. The same behavior is observed for P–0.26 system as well (see Supplementary Fig. S1). This suggests that both transitions in Trp-cage dynamics are collective phenomena, involving the concerted participation of all residues in the protein. Identical to the behavior of the whole Trp-cage protein, each residue shows a linear (harmonic) dependence of MSF on *T* at temperatures below the *T*_*D*_, with a slope change at *T*_*low*_. Furthermore, the MSF deviates from its harmonic behavior at *T*_*D*_. While all residues exhibit the transitions at the same temperatures (*T*_*low*_ and *T*_*D*_), the hydrophilic residues (e.g. K8, D9, and R16) exhibit larger-amplitude fluctuations at *T* >  *T*_*D*_, compared to the hydrophobic residues.

**Figure 7:**
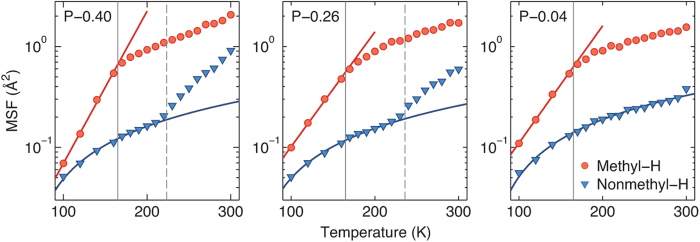
Average MSF of the hydrogen atoms in methyl and non-methyl groups for each system (note log scale for MSF). The gray solid and dashed lines represent *T*_*low*_ and *T*_*D*_, respectively. The red and blue lines show linear fits to *log*(MSF) vs. *T* of the methyl hydrogen atoms (*T* <  *T*_*low*_) and MSF vs. *T* of the non-methyl hydrogen atoms (*T* <  *T*_*D*_), respectively. The error bars are smaller than the symbol sizes.

One of the microscopic mechanisms that have been suggested to explain the transition at *T*_*low*_ is the activation of methyl group motions, whereby the MSF of the methyl-group hydrogen atoms exhibits an onset of anharmonic behavior^21^,^22^. In Fig. 7 we show the average MSF of the hydrogen atoms of the methyl and non-methyl groups. In Trp-cage, methyl groups are present in three residues: L2, I4, and L7. At temperatures below *T*_*low*_, the MSF of methyl-group hydrogen atoms increases exponentially with temperature. Instead of the *activation* of the methyl-group dynamics, which was suggested previously as a cause of the transition at *T*_*low*_^21^,^22^, the MSF of methyl-group hydrogens exhibits a transition from exponential to linear increase at *T*_*low*_. In contrast, the non-methyl hydrogen atoms exhibit a linear increase in the MSF without any transition at *T*_*low*_. Furthermore, we observe the opposite trend at *T*_*D*_: the non-methyl hydrogen atoms exhibit a deviation from linear MSF(*T*) behavior, while methyl-group hydrogen atoms exhibit no change in behavior at *T*_*D*_. This indicates that in Trp-cage powders the transition at *T*_*D*_ is correlated with enhanced dynamics of non-methyl groups, while a change in the temperature dependence of methyl group dynamics from exponential to linear occurs at *T*_*low*_.

## Discussion

We have investigated the dynamical transitions of Trp-cage powders with varying hydration levels (*h* =  0.40, 0.26, and 0.04 g/g). We identified two temperatures where transitions in the temperature-dependent protein dynamics occur. The average MSF of the protein heavy atoms increases linearly with temperature at low temperatures (100–150 K). The first transition at *T*_*low*_ (≈ 160 K) is characterized by a sudden change in the slope of this harmonic (linear) behavior. The second transition, PDT, occurs at a higher temperature (*T*_*D*_) where the MSF starts to depend nonlinearly upon temperature. We showed that the *T*_*D*_ can be precisely determined by locating the “calorimetric glass transition temperature” of the protein/water system, where a sudden jump in the heat capacity occurs. We find this heat-capacity jump to be more pronounced the larger the hydration level. The *T*_*low*_ and the MSF below *T*_*low*_ are identical for powder systems of different hydration levels. In contrast, dehydration shifts the *T*_*D*_ to a higher temperature, while greatly suppressing the protein dynamics at *T* >  *T*_*D*_.

As the powder systems are heated, the dynamics of methyl-group hydrogens, as measured by the MSF, increases exponentially with *T* up to *T*_*low*_, and linearly for *T* >  *T*_*low*_, without any signs of a transition at *T*_*D*_. In contrast, the MSF of non-methyl hydrogens increases linearly up to *T*_*D*_ and nonlinearly for *T* >  *T*_*D*_. This linear increase in the MSF of non-methyl hydrogens below *T*_*D*_ occurs without any slope change at *T*_*low*_. We have therefore identified distinct contributions from the methyl and non-methyl groups to the transitions at *T*_*low*_ and *T*_*D*_. Our findings on the temperature dependence of the methyl-group dynamics of the Trp-cage, however, are in contrast to previous studies^21^,^22^ where the *activation* of the methyl groups was suggested as the cause of the transition at *T*_*low*_. This suggests that important aspects of low-temperature protein dynamics may resist generalization across different individual proteins.

All 20 residues of the Trp-cage display the two transitions in their dynamics at the same *T*_*low*_ and *T*_*D*_ as the whole protein. The magnitude of their MSFs, however, depends largely on the degree of hydrophobicity/hydrophilicity of each residue. As the hydrated system (P–0.40) is heated, the characteristic motions of the hydrophilic residues are enhanced relative to those of the hydrophobic residues. The opposite is true for the dehydrated system (P–0.04) where the hydrophobic residues exhibit more pronounced increases in their MSFs. This points to hydration as an important process variable in modulating the temperature stability of solid-state pharmaceutical formulations.

We have presented a computational study of dynamical transitions in Trp-cage powders with varying hydration levels. It will be interesting to perform similar simulation studies with larger proteins or other biomolecules, such as RNAs and DNAs, in order to assess the generality of our findings. Another potentially fruitful avenue of inquiry will be to study a more complex protein matrix that consists not only of protein and water but includes also cosolutes, such as carbohydrates, that are commonly present in pharmaceutical formulations. By investigating the effects of sugar molecules on the residue-level dynamics of the simulated proteins, such studies would complement previous investigations on the shift of the dynamical transition temperatures due to the presence of sugar molecules^8^,^32^.

## Methods

### System Preparation

The powder system at a hydration level of 0.40 g/g was prepared by following the procedures described in detail in ref. 26. It contains 16 Trp-cages that are randomly translated and rotated, 771 water molecules, and 16 chloride ions. The net positive (+ 1*e*) charge of each Trp-cage at neutral pH was balanced by the negatively charged chloride ion (− 1*e*). Powders at lower hydration levels were prepared by dehydrating this powder, through cycles comprising the removal of one water molecule and the relaxation of the resulting system through 200 ps of NPT MD simulation at 300 K and 1 bar^26^. The water molecule to be removed was randomly chosen and accepted/rejected by standard Metropolis criteria based on the associated Boltzmann factor (*e*^−*β*Δ*U*^), where *β* is the inverse of the product of Boltzmann’s constant and temperature, and Δ *U* is the change in configurational energy that would result from removing the water molecule in question. In this way, water molecules that are more strongly bound to protein units are less likely to be removed, preventing a potentially large perturbation to the protein structure.

### Molecular Dynamics Simulation

The GROMACS^33^,^34^,^35^,^36^ package was used for all MD simulations. The leap-frog algorithm was used to integrate the equations of motion, with a time step of 1 fs. Temperature and pressure were controlled using Nosé-Hoover thermostat^37^,^38^ with a 0.1 ps time constant and the Parrinello-Rahman barostat^39^,^40^ with a 1 ps time constant, respectively. Anisotropic pressure coupling ensured that fluctuations in the dimensions of the orthorhombic simulation box were independent of each other. Periodic boundary conditions were applied in all three dimensions. We truncated the short-range interactions at 1 nm and applied the standard long-range dispersion corrections for the energy and pressure^41^. The reciprocal part of the Ewald sum for long-range electrostatics was calculated using the smooth particle mesh Ewald method^42^. The linear constraint solver algorithm (LINCS)^43^,^44^ and SETTLE^45^ were used to constrain all bonds in the protein and water molecules, respectively. Proteins and water molecules were modeled using Amber ff03w^46^,^47^ and TIP4P/2005^48^ force fields, respectively.

The systems were equilibrated at 300 K and 1 bar initially, through 5 ns of NVT MD, followed by 5 ns of NPT MD. Each prepared system was replicated and equilibrated to low temperatures (100–300 K) by performing an NPT MD simulation during which the temperature was decreased linearly from 300 K to the temperature of interest at a rate of 4 K/ns. Variations of this cooling rate (1, 4, and 10 K/ns) were tested for one of the powders (*h* =  0.40 g/g), and identical protein dynamics were observed. We then performed 200 ns of NPT MD and used the last 150 ns of the trajectory for analysis. We performed the block-averaging analysis^49^,^50^ by dividing each trajectory into 5 blocks, in order to estimate the standard error for each observable we analyzed.

### Mean-Square Fluctuation

In order to compute the mean-square fluctuation (MSF), the structure of each protein (using protein heavy atoms) at all time steps was first aligned to the structure of the corresponding protein at time =  0. The MSF of each protein heavy atom, *i*, was then calculated by computing the variance in its atomic positions from the average position:


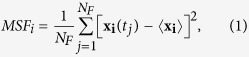


where *N*_*F*_ is the number of frames in a trajectory, **x**_**i**_ is the position of an atom *i*, and 〈 〉 is the ensemble average. The atomic MSF values were then mass-averaged to compute the MSF for each Trp-cage, and the MSF of the individual Trp-cages were then averaged to yield the average MSF at each temperature.

## Additional Information

**How to cite this article**: Kim, S. B. *et al.* Computational investigation of dynamical transitions in Trp-cage miniprotein powders. *Sci. Rep.* **6**, 25612; doi: 10.1038/srep25612 (2016).

## Supplementary Material

Supplementary Information
