# Supplementary material for: Computational investigation of dynamical transitions in Trp-cage miniprotein powders
Source: Sci Rep. 2016 May 6;6:25612. doi: 10.1038/srep25612 (PMC4858699; doi:10.1038/srep25612)
Supplement: Supplementary Information [file srep25612-s1.pdf]

Supplementary Information

Computational investigation of dynamical transitions in Trp-cage miniprotein powders

Sang Beom Kim[1], Devansh R. Gupta[1], and Pablo G. Debenedetti[1,*]

[1]*Department of Chemical and Biological Engineering, Princeton University, Princeton, New Jersey 08544, United States*

*Corresponding author: pdebene@princeton.edu

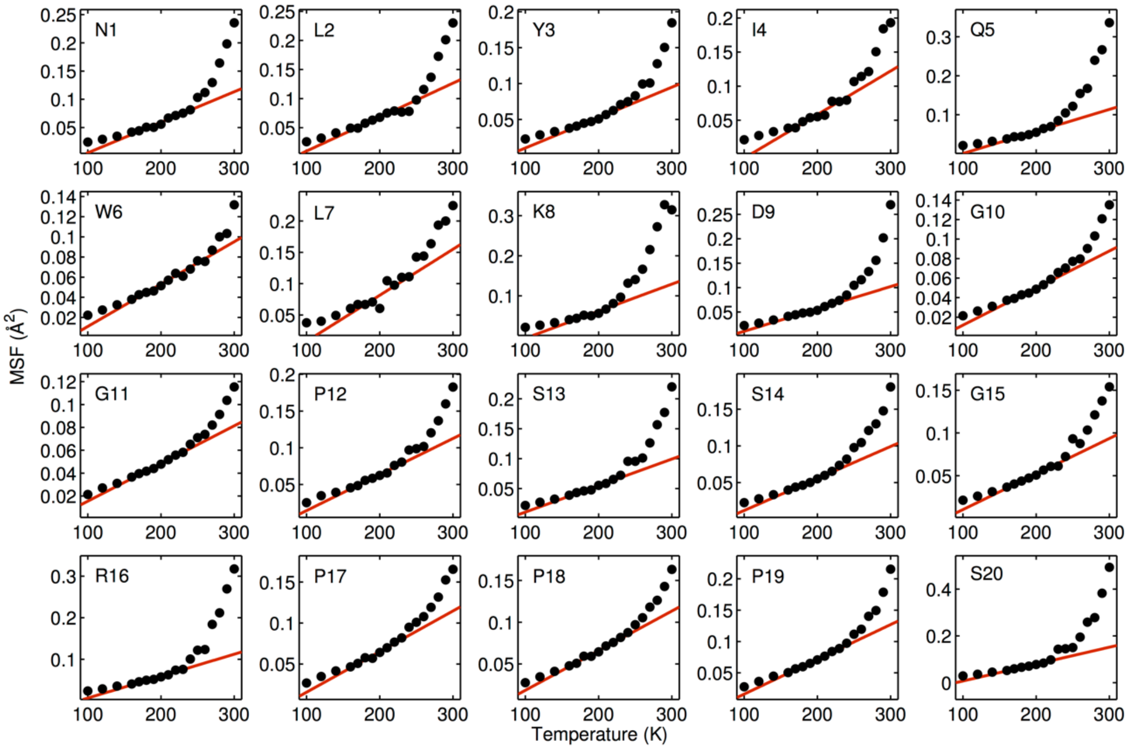

Figure S1. Average MSF of individual residues in Trp-cage (P–0.26 system). The red line depicts the linear fit to the data between temperatures T_{low} and T_D (160K and 236K, respectively). The error bars are smaller than the symbol sizes.
